# Moderators of design and delivery component effects on engagement with a digital parenting intervention: evidence from a factorial randomized trial in Tanzania

**DOI:** 10.1093/abm/kaag033

**Published:** 2026-06-21

**Authors:** Roselinde Janowski, Yulia Shenderovich, Joyce Wamoyi, David Stern, Jamie M Lachman, G J Melendez-Torres, Lucie D Cluver

**Affiliations:** Department of Social Policy and Intervention, University of Oxford, Oxford, United Kingdom; Centre for Development, Evaluation, Complexity, and Implementation in Public Health Improvement (DECIPHer), School of Social Sciences, Cardiff University, Cardiff, United Kingdom; Wolfson Centre for Young People’s Mental Health, Cardiff University, Cardiff, United Kingdom; National Institute for Medical Research, Mwanza Research Centre, Mwanza, Tanzania; Innovations in Development, Education, and the Mathematical Sciences (IDEMS) International, Reading, United Kingdom; Department of Social Policy and Intervention, University of Oxford, Oxford, United Kingdom; Centre for Social Science Research, University of Cape Town, Cape Town, South Africa; Parenting for Lifelong Health, Oxford, United Kingdom; Faculty of Health and Life Sciences, University of Exeter, Exeter, United Kingdom; Department of Social Policy and Intervention, University of Oxford, Oxford, United Kingdom; Department of Psychiatry and Mental Health, University of Cape Town, Cape Town, South Africa

**Keywords:** digital health, engagement, moderators, parenting, factorial experiment, Multiphase Optimization Strategy, Tanzania

## Abstract

**Background:**

Digital health interventions are rapidly expanding in low- and middle-income countries (LMICs), yet little is known about how design and delivery components can be tailored to support engagement across diverse populations. Addressing this gap is critical for effective and equitable implementation.

**Purpose:**

This study used a factorial experiment, embedded within the Optimization Phase of the Multiphase Optimization Strategy (MOST), to examine whether caregiver characteristics moderated the effects of design and delivery components on engagement with an app-based parenting intervention in Tanzania.

**Methods:**

A 2 × 2 × 2 cluster-randomized factorial experiment was conducted in Mwanza, Tanzania (16 clusters; 614 caregivers of adolescents). Three components were tested: guidance (guided vs self-guided), app design (unstructured vs structured), and digital support (enhanced vs basic). Engagement was operationalized as the number of intervention modules completed, which was tracked automatically via the app. Generalized linear mixed-effects models examined moderation by caregiver gender, age, financial stress, food insecurity, positive parenting, child maltreatment, and caregiver depression.

**Results:**

Gender, age, positive parenting, and depressive symptoms moderated the effects of specific components on engagement. Women showed significantly higher engagement with the unstructured versus structured app design (incidence rate ratio [IRR] = 1.48, 95% CI, 1.23-1.80), whereas no significant app design differences were observed among men. Older caregivers showed greater engagement under guided delivery (vs self-guided; IRR = 1.15, 95% CI, 1.06-1.25) and enhanced digital support (vs basic support; IRR = 1.15, 95% CI, 1.06-1.26). Greater engagement under guided versus self-guided delivery was also observed among caregivers reporting more positive parenting practices (IRR = 1.03, 95% CI, 1.02-1.04) and higher depressive symptoms (IRR = 1.06, 95% CI, 1.03-1.09). However, caregivers reporting more positive parenting practices showed lower engagement under enhanced digital support (vs basic support; IRR = 0.96, 95% CI, 0.95-0.98). No moderation effects were observed for financial stress, food insecurity, or child maltreatment.

**Conclusions:**

Tailoring design and delivery components to specific caregiver characteristics may enhance engagement and promote more equitable implementation of digital parenting interventions in LMICs.

**Clinical trial registration number:**

The trial was pre-registered on the Pan-African Clinical Trial Registry PACTR202210657553944; https://pactr.samrc.ac.za/TrialDisplay.aspx?TrialID=24051.

## Introduction

Violence against children is a major contributor to the global burden of disease, affecting over 1 billion children each year.^1^ Parenting interventions are among the most effective strategies for preventing violence globally.^2^ However, scaling these interventions remains constrained by high delivery costs and barriers to access, particularly among families experiencing adversity, marginalization, and socioeconomic stress.^3–5^ Digital delivery offers a promising solution to these challenges, with growing evidence indicating comparable effectiveness to in-person parenting programs.^6–9^

Parenting for Lifelong Health for Parents and Teens (PLH Teens) is a group-based parenting intervention designed to reduce adolescents’ exposure to violence by strengthening positive parenting, improving caregiver–child communication, and promoting caregiver well-being.^10^ PLH Teens is one of the few low-cost parenting interventions for families with adolescents that has been rigorously tested and disseminated in low- and middle-income countries (LMICs).^11^ However, in-person delivery remains resource-intensive and logistically challenging to scale. To address these barriers, ParentApp was developed to deliver a remote, app-based version of PLH Teens.

Digital parenting interventions such as ParentApp frequently face challenges with engagement, broadly defined as the amount, frequency, duration, and depth of program use.^12^ These challenges may be particularly pronounced in LMICs, where digital infrastructure gaps and socioeconomic constraints can limit uptake and sustained use.^13^ Greater engagement in parenting interventions has consistently been associated with stronger outcomes,^14^^,^^15^ making engagement optimization essential for achieving public health impact. However, little is known about which implementation strategies are most effective for engaging different caregiver subgroups. Identifying for whom specific strategies are most effective is therefore critical for reducing inequities in who engages with, benefits from, and is ultimately reached by digital parenting interventions.

In implementation science, implementation strategies are defined as “methods or techniques used to enhance the ­adoption, implementation, and sustainability of a clinical program or practice” [p. 2].^16^ In digital interventions, implementation strategies may include design components, which determine how content is organized and accessed within the platform, and delivery components, which determine how the intervention is delivered and supported in practice. The Multiphase Optimization Strategy (MOST) provides a framework for evaluating these design and delivery components.^17^^,^^18^ In the Optimization Phase of MOST, factorial randomized experiments are typically used to assess the individual and combined effects of selected components and can additionally be used to examine interactions between components and participant characteristics.

In Tanzania, Janowski et al^19^ conducted a factorial experiment to optimize engagement with ParentApp by testing 3 design and delivery components while holding the core parenting content constant. The components included guidance (self-guided app use versus app use with facilitator-moderated WhatsApp groups), app design (sequential weekly module release with generic illustrations versus immediate unrestricted module access with culturally adapted illustrations), and digital support (basic onboarding versus enhanced smartphone literacy training). Main trial findings showed that guided delivery and an unstructured app design significantly increased engagement, whereas enhanced digital support offered no additional benefit.^19^

However, these findings reflect average effects and may mask important differences in how caregiver subgroups respond to specific components. Moderator analyses examine for whom or under what conditions particular components are most effective,^20^ helping to identify differential responses and inform more targeted and equitable implementation. Unlike predictors, which are associated with outcomes irrespective of intervention assignment, moderators interact with intervention components to alter the strength or direction of effects.^20^

To date, few studies of digital parenting interventions have examined moderators, and those that have typically focused on overall program effects in randomized controlled trials (RCTs)^21^ or meta-analyses,^6^^,^^22^^,^^23^ rather than on the effects of discrete intervention components. As a result, little is known about how specific components influence engagement across caregiver subgroups. The current study addresses this gap by examining whether baseline caregiver characteristics moderate the effects of specific design and delivery components on engagement with ParentApp.

Candidate moderators were selected based on contextual relevance and established person-level associations with engagement in digital interventions. Demographic characteristics such as gender and age were included because they are key determinants of digital engagement, influencing mobile phone ownership, internet access, and confidence in using technology.^24^^,^^25^ These disparities are often more pronounced in LMICs, where women and older adults tend to have lower device access and connectivity,^26^ potentially shaping both overall engagement and responsiveness to strategies aimed at improving engagement.

Socioeconomic characteristics were included based on prior evidence linking economic strain to lower engagement in digital parenting programs.^22^^,^^27–30^ Financial stress and food insecurity were examined as complementary indicators of socioeconomic adversity, capturing both subjective economic pressure and material hardship. Economic constraints may limit caregivers’ ability to engage with digital interventions, as well as shape how they respond to design and delivery components intended to support engagement.

Positive parenting and child maltreatment were examined as candidate moderators because both are primary behavioral targets of ParentApp, and engagement is a key mechanism through which parenting interventions produce change.^14^^,^^15^ Baseline levels of these constructs may therefore influence how caregivers respond to specific design and delivery components. In the original PLH Teens RCT in South Africa, Shenderovich et al^31^ examined positive parenting and child maltreatment as moderators of treatment outcomes and found no significant effects. However, to our knowledge, no study has examined whether these constructs moderate engagement with specific design and delivery components. Prior digital parenting research has nonetheless linked positive parenting practices to both higher^30^ and lower^32^ engagement, while harsh and abusive parenting behaviors have been associated with lower engagement.^33^ These findings suggest that the relationship between baseline parenting behaviors and engagement may depend on how interventions are designed and delivered.

Finally, caregiver depressive symptoms have been associated with lower engagement in digital interventions, potentially due to low mood, fatigue, and reduced motivation.^25^ However, more severe symptoms have also been associated with greater perceived need for support and willingness to engage,^25^ suggesting that the relationship between depressive symptoms and engagement may depend on delivery context. Consistent with these findings, prior research found that higher depressive symptoms in a digital parenting intervention were associated with lower enrollment in self-directed online delivery, but not in telehealth formats involving human support.^34^ To our knowledge, no study has examined whether depressive symptoms moderate engagement with specific design and delivery components, and given that caregiver wellbeing is also a key outcome of ParentApp, baseline depressive symptoms were included as a candidate moderator.

Accordingly, this study examines whether caregiver characteristics across demographic (gender, age), socioeconomic (financial stress, food insecurity), and psychosocial (positive parenting, child maltreatment, caregiver depression) domains moderate the effects of design and delivery components on engagement with ParentApp. Given the exploratory nature of this analysis in a novel LMIC context, we did not specify a priori hypotheses regarding individual moderators. By identifying for whom specific components are most effective, this study aims to inform more targeted and equitable implementation of digital parenting interventions in LMICs.

## Methods

### Experimental design

This study uses data from the ParentApp optimization trial, a cluster-randomized full factorial experiment conducted across 16 low-income peri-urban and urban communities in Mwanza, Tanzania.^19^ The trial tested 3 components: guidance (guided vs self-guided), app design (unstructured vs structured), and digital support (enhanced vs basic). Component selection was informed by prior feasibility testing, which identified key barriers to engagement with ParentApp.^35^ Full trial procedures and primary findings are detailed elsewhere.^19^^,^^35^ The current study presents a follow-up analysis examining whether caregiver characteristics moderated the effects of design and delivery components on engagement.

### Participants

Participants were parents or primary caregivers of adolescents aged 10-17 years. Eligibility criteria included being 18 years or older, co-residing with their adolescent for at least 4 nights per week, and having access to an Android smartphone (operating system 5.1.1 or higher) to use ParentApp.

### Intervention

All participants received ParentApp, a noncommercial smartphone app designed for offline use. The app was adapted from the PLH Teens program^10^ and consisted of 12 modules: an introductory module on caregiver self-care (which included the baseline assessment), 10 core modules focused on evidence-based parenting practices to promote positive parenting and reduce harsh discipline, and a final module that reviewed key concepts and included a post-intervention assessment. Full intervention details, including the TIDieR Checklist, are available in Janowski et al^19^

### Experimental components

#### Guidance (self-guided vs guided)

Clusters were randomized to either self-guided delivery or guided delivery involving facilitator-moderated WhatsApp groups. Each cluster assigned to the guided condition was linked to a dedicated WhatsApp group overseen by a mixed-gender facilitator pair (8 groups total, 4 facilitator pairs). These groups were intended to promote social connection and sustain engagement with ParentApp throughout the 12-week intervention period. Following a standardized manual, facilitators posted weekly reminders and discussion prompts, monitored discussions, and responded to participants’ questions. Facilitators also conducted weekly 1-hour live chat sessions to encourage peer interaction and discussion.

#### App design (structured vs unstructured)

Clusters were randomized to either a structured or unstructured app design. ParentApp was originally designed with sequential weekly module release to align with PLH Teens’ learning objectives and pacing and featured abstract, gender-neutral illustrations intended to support cross-cultural scalability. Although user testing and piloting indicated that this version was broadly acceptable, 2 potential areas for improvement were identified: some caregivers preferred greater flexibility in accessing content, while others raised concerns about the acceptability of the generic illustrations. In response, the trial evaluated 2 app designs. The structured version maintained sequential module delivery over 12 weeks and retained the original generic illustrations. In the unstructured version, all modules were available immediately upon installation, allowing caregivers to navigate content at their own pace, and the generic illustrations were replaced with culturally adapted imagery tailored to the Tanzanian context.

#### Digital support (basic vs enhanced)

All clusters received basic digital support consisting of an embedded app tutorial and a group-based demonstration of key app features during onboarding. Clusters assigned to the enhanced support condition received an additional 15-20-minute training session focused on improving general smartphone literacy and confidence in broader smartphone use.

### Procedures

Onboarding sessions were conducted between October 24 and December 1, 2022, in schools and community centers. Following cluster-level randomization, community leaders within each cluster invited eligible caregivers to attend these sessions. One onboarding session was held per cluster (16 in total), with additional sessions conducted where recruitment targets were not met. Sessions were delivered by research staff from the National Institute for Medical Research (NIMR) and facilitators from Investing in Children and Strengthening Their Societies (ICS).

Each onboarding session included: (1) study information and eligibility screening, (2) informed consent, (3) app onboarding and digital support training, and (4) baseline data collection. Following consent, caregivers were divided into small groups of 5-10 participants to facilitate hands-on support with app installation and navigation. NIMR staff guided participants through downloading and installing ParentApp (available via Google Play), after which all participants received basic digital support training and completed the app’s introductory module, which included the embedded baseline assessment. Sessions lasted approximately 90 minutes. In clusters assigned to enhanced digital support, caregivers received an additional 15-20-minute group-based smartphone literacy training.

To enable data synchronization, participants were provided with 1 GB of mobile data per month throughout the intervention period. Participants also received US $2 as compensation for attending the onboarding session. A CONSORT flow diagram detailing cluster allocation and participant progression through the study is provided in Supplementary Figure S1.

### Measures

#### Engagement outcome

Engagement was measured as the number of modules completed, a widely used indicator of sustained engagement in digital parenting interventions.^36^ Module completion was defined as navigating all pages within a module and selecting “Finish” on the final page. Each completed module was scored as 1, yielding a total engagement score ranging from 0 to 12, with higher scores indicating greater exposure to intervention content. Other engagement metrics reported in the main trial paper (eg, time spent using the app, home practice activity reviews, and behavior logs) were not examined due to data quality concerns.^19^

#### Candidate moderators

All candidate moderators were drawn from open-access instruments recommended by the Global Parenting Initiative (GPI) and previously used in large-scale PLH program implementation in Tanzania and other LMICs.^37^ Prior to the trial, measures underwent cultural adaptation, including co-development with Tanzanian experts in violence against children, forward–backward translation into Kiswahili, and pilot testing with 100 Tanzanian families. Abbreviated scales were intentionally selected to minimize participant burden during app-based onboarding, reflecting the study’s pragmatic design and focus on engagement optimization rather than comprehensive caregiver outcome assessment.

Demographic moderators included caregiver gender (woman/man) and age (years). Additional demographic variables collected but not included in the present analyses were household composition and adolescent orphanhood status. Socioeconomic factors were assessed using 2 single items adapted from the Financial Self-Efficacy Scale.^38^ Financial stress was measured using the item, “How many times in the past month have you felt worried or anxious about money?”, rated on a 0 to ≥8 frequency scale. Food insecurity was assessed as the number of days in the past month the household ran out of money for food (0-30 days). These items were analyzed separately due to differences in response formats.

Positive parenting was measured using 5 items from subscales of the Alabama Parenting Questionnaire.^39^ Caregivers reported the frequency of specific parenting behaviors toward their adolescents on a 9-point count scale (0 to ≥8 times). The measure included items assessing positive parenting (eg, “How many times in the past month have you praised your teen?”), involvement (eg, “How many times in the past month did you get involved in activities that your teen likes?”), and supervision (eg, “How many times in the past month did your teen stay out in the evening past the time they were supposed to be at home?”). Responses were summed to create a total positive parenting score, with higher scores indicating greater positive parenting. Internal consistency in the present sample was modest (α = .54), likely reflecting the brevity of the scale.

Child maltreatment (physical and emotional abuse) was measured using 4 items from the International Society for Prevention of Child Abuse and Neglect Screening Tool-Trial Version.^40^ Caregivers reported the frequency of behaviors in the past month on a 9-point count scale (0 to ≥8 times). The measure included items assessing physical abuse (eg, “How many times in the past month did you physically discipline your teen by hitting, spanking, or slapping with your hand or an object like a stick or belt?”) and emotional abuse (eg, “How many times in the past month did you shout, scream, or yell at your teen?”). Responses were summed to create a total child maltreatment score, with higher scores indicating greater maltreatment. Internal consistency in the present sample was good (α = .76).

Caregiver depression was measured using 3 items from the Center for Epidemiological Studies Depression Scale.^41^ Caregivers reported the number of days in the past week they had: (1) felt depressed, (2) felt that everything they did was an effort, and (3) felt hopeful about the future (reverse-coded). Responses were rated on a 0-7 day frequency scale and summed, with higher scores indicating greater depressive symptoms. Internal consistency in the present sample was modest (α = .50).

### Statistical analyses

Missing data were addressed using multiple imputation by chained equations (MICE) under a missing-at-random assumption.^42^ Ten imputed datasets were generated using predictive mean matching. The predictor matrix included all complete engagement variables, baseline variables, experimental factors, and cluster allocation. Imputations were conducted within clusters to preserve the clustered data structure. Behavioral items contributing to total scale scores were imputed at the item level prior to aggregation. Subsequent analyses followed an intention-to-treat approach, with estimates pooled across imputations using Rubin’s rules.

**Figure 1:**
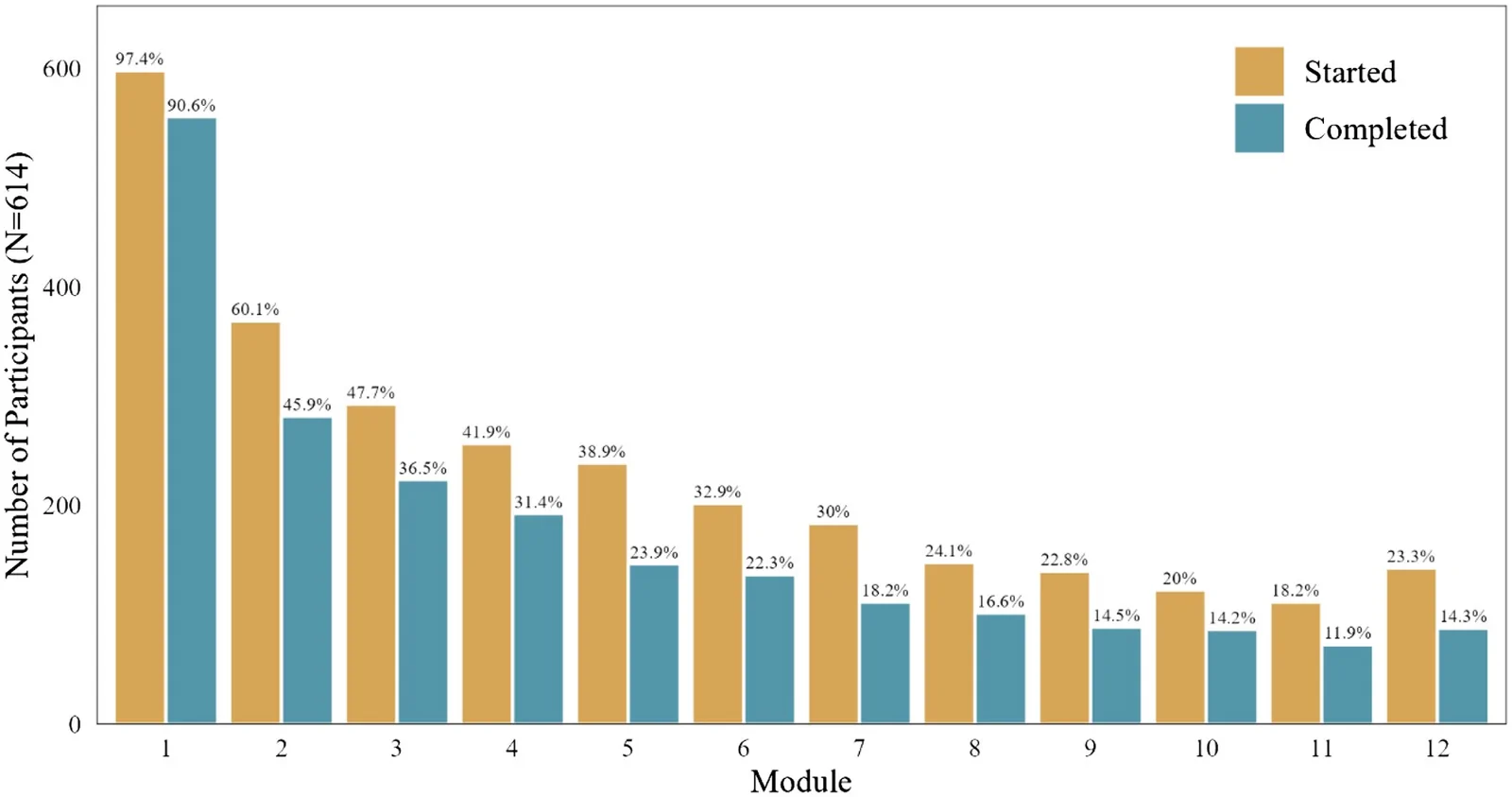
Percentage of participants who started and completed each module.

Generalized linear mixed-effects models were fitted to account for the nested data structure, specifying a Poisson distribution for the count outcome. Analyses proceeded in 2 stages for each candidate moderator. In step 1, separate models were estimated for each component and moderator to assess their independent associations with engagement. In step 2, moderation was tested by adding an ­interaction term between the component and moderator (component × moderator) to the models from step 1. Likelihood ratio tests were used to assess the significance of interaction effects. To separate individual- and cluster-level effects, moderators were cluster-mean centered. The cluster-level mean of each tested moderator was also included in the corresponding model to account for within- and between-cluster variation. All models additionally adjusted for caregiver age and gender as a priori demographic covariates, both of which were sample-mean centered. To avoid model non-convergence, caregiver age was divided by a factor of 10. Component variables were effect coded (−1, 1), and resulting unstandardized regression coefficients and SEs were multiplied by a scaling constant of 2.^43^ Incidence rate ratios (IRRs) were derived by exponentiating coefficients and represent the relative change in module completion associated with a one-unit increase in the moderator. To control the false discovery rate (FDR) associated with multiple comparisons, the Benjamini–Hochberg procedure^44^ was applied to the *P* values from likelihood ratio tests comparing models with and without the interaction terms. *P* values are reported in the text and tables, but statistical significance was determined using thresholds derived from the Benjamini–Hochberg procedure. All analyses were conducted in R (Version 4.3.1).

## Results

### Overview of participants and engagement

Table 1 summarizes baseline demographic, socioeconomic, and psychosocial characteristics. A total of 614 caregivers (33.4% men) were included in the analyses, ranging in age from 18 to 75 years (mean = 35.94, SD = 11.84). Using cluster-level randomization, caregivers were assigned to guided (*n* = 284) or self-guided (*n* = 330) delivery, structured (*n* = 332) or unstructured (*n* = 282) app design, and enhanced (*n* = 319) or basic (*n* = 295) digital support.

**Table 1:** Baseline characteristics of study participants.

Continuous measures	*n*	*M*	*SD*	Range
**Age (years)**	577	35.94	11.84	18-75
**Financial stress**	574	4.27	2.75	0-8
**Food insecurity**	576	7.30	7.78	0-30
**Positive parenting**	614	10.89	6.78	0-40
**Child maltreatment**	614	6.62	6.42	0-32
**Caregiver depression**	614	8.29	3.95	0-21
**Categorical measure**	***n***	**%**		
**Gender (men)**	205	33.4		

Sample sizes vary due to missing data.

Caregivers reported high levels of socioeconomic adversity: 23% (132/574) reported feeling worried or anxious about money for 7 or more days in the past month, and 84.2% (485/576) reported running out of money to pay for food at least once in the past month. Nearly half of caregivers (47.9%) reported that their adolescent had experienced the death of a caregiver during their lifetime. The relationship between caregivers and adolescents (eg, biological parent or other relative) was not systematically recorded. Detailed participant characteristics and full engagement descriptives are reported elsewhere.^19^

Figure 1 shows the number of participants who began and completed each module. Engagement declined over time, with 38.4% of caregivers completing at least 25% of modules, 21.5% completing at least 50%, 13.8% completing at least 75%, and 8% completing all 12 modules.

### Moderators of component effects on engagement

**Figure 2:**
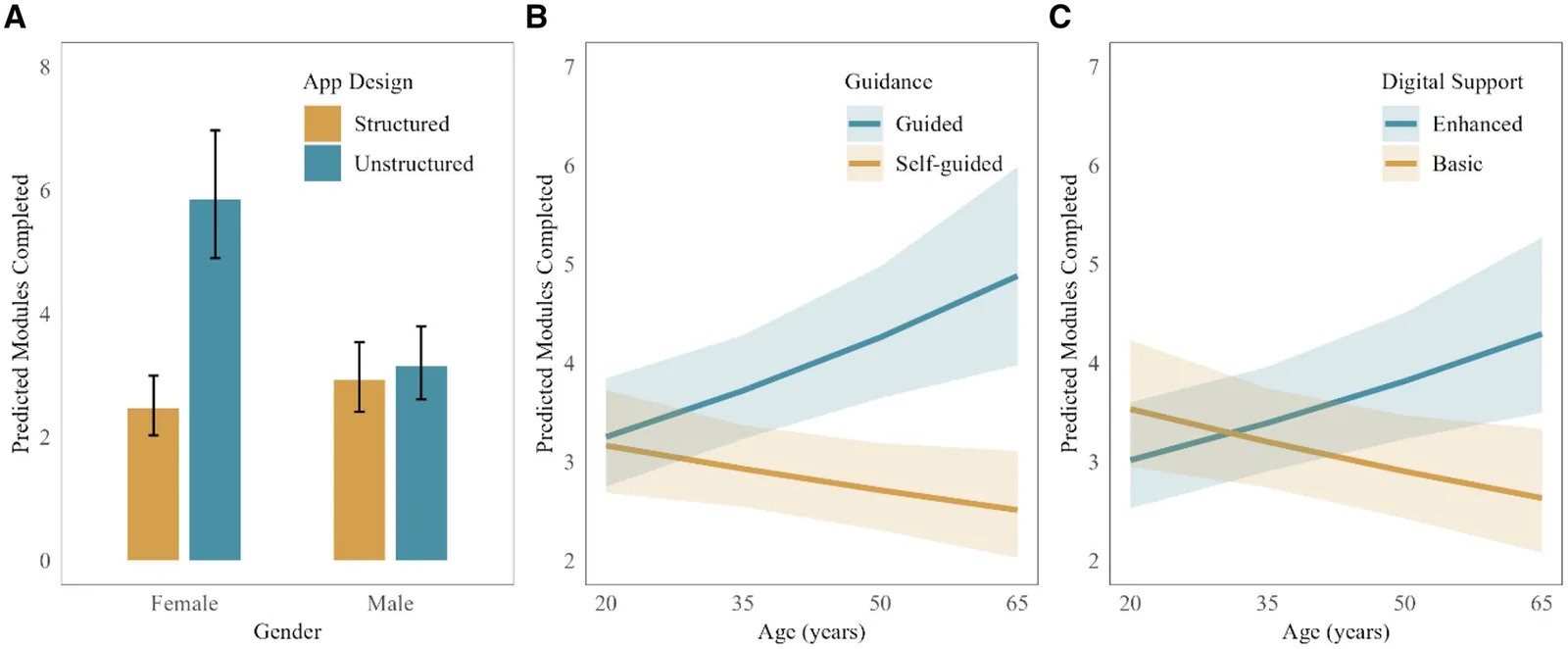
Moderation effects of caregiver gender and age on predicted modules completed. (A) Gender × app design. (B) Age × guidance. (C) Age × digital support. Error bars and shaded areas represent 95% confidence intervals.

**Figure 3:**
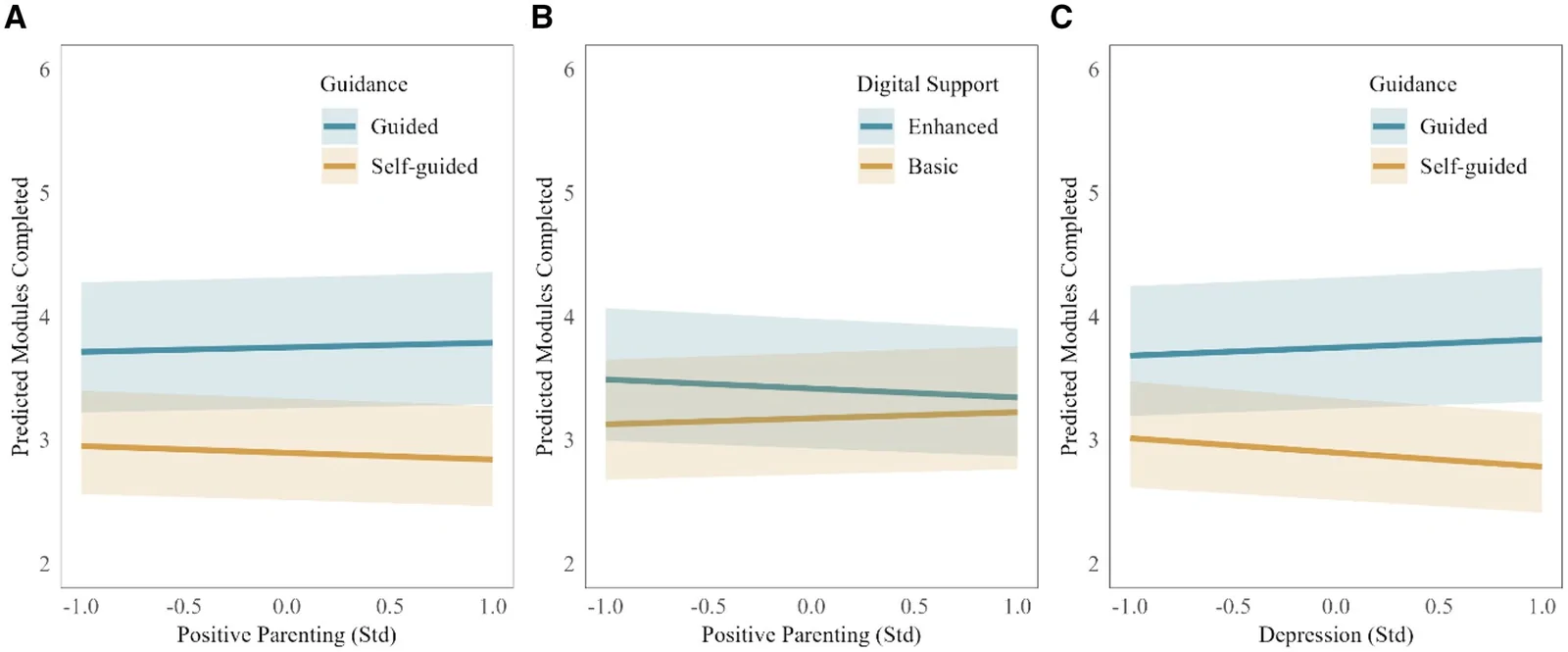
Moderation effects of positive parenting and caregiver depression on predicted modules completed. (A) Positive parenting × guidance. (B) Positive parenting × digital support. (C) Depression × guidance. Shaded areas represent 95% confidence intervals.

Six significant moderation effects were identified across demographic (Table 2; Figure 2) and psychosocial (Table 3; Figure 3) characteristics following Benjamini–Hochberg correction. No significant moderation effects were observed for socioeconomic characteristics (Table 4).

**Table 2:** Age and gender as moderators of component effects on engagement.

	Gender				Age			
Potential moderator	*β*	IRR	95 % CI	*P*	*β*	IRR	95% CI	*P*
**Guidance**								
**Step 1**								
** Guided vs self-guided**	0.13	1.29	[1.07-1.56]	.007	0.13	1.29	[1.06-1.57]	.011
** Potential moderator**	0.14	1.33	[1.10-1.61]	.004	0.02	1.04	[0.95-1.13]	.400
**Step 2**								
** Guided vs self-guided**	0.13	1.29	[1.07-1.55]	.009	0.13	1.29	[1.06-1.57]	.012
** Potential moderator**	0.14	1.33	[1.10-1.62]	.003	0.02	1.04	[0.95-1.13]	.384
** Interaction term**	0.10	1.21	[1.00-1.47]	.047	0.07	1.15	[1.06-1.25]	.001
**Likelihood ratio test**				.047				**.001** ^a^
**App design**								
**Step 1**								
** Unstructured vs structured**	0.24	1.61	[1.31-1.98]	<.001	0.20	1.50	[1.26-1.79]	<.001
** Potential moderator**	0.15	1.34	[1.10-1.62]	.003	0.02	1.04	[0.96-1.13]	.362
**Step 2**								
** Unstructured vs structured**	0.23	1.60	[1.30-1.96]	<.001	0.20	1.50	[1.26-1.78]	<.001
** Potential moderator**	0.11	1.25	[1.03-1.52]	.021	0.02	1.04	[0.95-1.13]	.410
** Interaction term**	0.20	1.48	[1.23-1.80]	<.001	0.03	1.05	[0.96-1.15]	.252
**Likelihood ratio test**				**<.001** ^a^				.255
**Digital support**								
**Step 1**								
** Enhanced vs basic**	0.05	1.11	[0.90-1.37]	.309	0.04	1.08	[0.86-1.34]	.512
** Potential moderator**	0.14	1.33	[1.10-1.61]	.003	0.02	1.04	[0.95-1.13]	.393
**Step 2**								
** Enhanced vs basic**	0.05	1.12	[0.91-1.38]	.302	0.04	1.07	[0.86-1.34]	.520
** Potential moderator**	1.14	1.33	[1.10-1.62]	.003	0.01	1.01	[0.93-1.11]	.772
** Interaction term**	−0.03	0.95	[0.79-1.15]	.601	0.07	1.15	[1.06-1.26]	.001
**Likelihood ratio test**				.602				**.002** ^a^

Dependent variable = number of modules completed. All models adjusted for age and gender. IRR = exp(2 * β), where β represents the unstandardized regression coefficient from effect-coded experimental factors. Likelihood ratio test *P* values were adjusted for multiple comparisons using the Benjamini–Hochberg procedure.

a Bold *P* values indicate significance after Benjamini–Hochberg correction (gender × app design, *P* = .004; age × guidance, *P* = .004; age × digital support, *P* = .007).

**Table 3:** Baseline psychosocial characteristics as moderators of component effects on engagement.

	Positive parenting				Child maltreatment				Parental depression			
Potential moderator	*β*	IRR	95 % CI	*P*	*Β*	IRR	95% CI	*P*	*β*	IRR	95% CI	*P*
**Guidance**												
**Step 1**												
** Guided vs self-guided**	0.14	1.31	[1.08-1.60]	.007	0.06	1.13	[0.96-1.34]	.146	0.15	1.35	[1.07-1.70]	.010
** Potential moderator**	−0.00	1.00	[0.98-1.01]	.560	−0.00	1.00	[0.98-1.01]	.976	−0.01	0.98	[0.95-1.01]	.238
**Step 2**												
** Guided vs self-guided**	0.14	1.31	[1.08-1.60]	.007	0.06	1.13	[0.95-1.33]	.159	0.15	1.35	[1.07-1.71]	.010
** Potential moderator**	−0.00	0.99	[0.98-1.01]	.274	−0.00	1.00	[0.98-1.01]	.777	−0.01	0.98	[0.95-1.01]	.142
** Interaction term**	0.01	1.03	[1.02-1.04]	<.001	0.01	1.02	[1.00-1.03]	.023	0.03	1.06	[1.03-1.09]	<.001
**Likelihood ratio test**				**<.001** ^a^				.025				**<.001** ^a^
**App design**												
**Step 1**												
** Unstructured vs structured**	0.20	1.50	[1.26-1.79]	<.001	0.14	1.32	[1.12-1.55]	.001	0.20	1.49	[1.27-1.75]	<.001
** Potential moderator**	−0.00	1.00	[0.98-1.01]	.545	−0.00	1.00	[0.98-1.01]	.945	−0.01	0.98	[0.95-1.01]	.219
**Step 2**												
** Unstructured vs structured**	0.20	1.50	[1.26-1.79]	<.001	0.14	1.32	[1.13-1.55]	.001	0.20	1.49	[1.27-1.75]	<.001
** Potential moderator**	−0.00	0.99	[0.98-1.01]	.441	0.00	1.00	[0.99-1.02]	.945	−0.01	0.98	[0.95-1.01]	.183
** Interaction term**	0.00	1.01	[0.99-1.02]	.338	−0.00	0.99	[0.98-1.01]	.495	−0.02	0.97	[0.94-1.00]	.026
**Likelihood ratio test**				.239				.501				.027
**Digital support**												
**Step 1**												
** Enhanced vs basic**	0.05	1.11	[0.89-1.38]	.358	0.02	1.04	[0.89-1.22]	.616	0.05	1.10	[0.88-1.37]	.419
** Potential moderator**	−0.00	1.00	[0.98-1.01]	.565	−0.00	1.00	[0.98-1.01]	.971	−0.01	0.98	[0.95-1.01]	.234
**Step 2**												
** Enhanced vs basic**	0.05	1.11	[0.89-1.37]	.361	0.02	1.04	[0.89-1.22]	.598	0.05	1.10	[0.88-1.37]	.424
** Potential moderator**	−0.00	0.99	[0.98-1.01]	.497	0.00	1.00	[0.99-1.02]	.976	−0.01	0.98	[0.95-1.01]	.256
** Interaction term**	−0.02	0.96	[0.95-0.98]	<.001	−0.01	0.99	[0.97-1.00]	.091	−0.01	0.98	[0.95-1.01]	.172
**Likelihood ratio test**				**<.001** ^a^				.096				.173

Dependent variable = number of modules completed. All models adjusted for age and gender. IRR = exp(2 * β), where β represents the unstandardized regression coefficient from effect-coded experimental factors. Likelihood ratio test *P* value were adjusted for multiple comparisons using the Benjamini–Hochberg procedure.

a Bold *P* values indicate significance after Benjamini–Hochberg correction (positive parenting × guidance, *P* = .004; positive parenting × digital support, *P* = .004; parental depression × guidance, *P* = .004).

**Table 4:** Socioeconomic factors as moderators of component effects on engagement.

	Financial stress				Food insecurity			
Potential moderator	*β*	IRR	95 % CI	*P*	*β*	IRR	95% CI	*P*
**Guidance**								
**Step 1**								
** Guided vs self-guided**	0.13	1.31	[1.05-1.63]	.019	0.13	1.29	[1.05-1.58]	.015
** Potential moderator**	0.02	1.03	[1.00-1.07]	.066	0.00	1.00	[0.99-1.02]	.696
**Step 2**								
** Guided vs self-guided**	0.13	1.30	[1.04-1.63]	.020	0.13	1.29	[1.05-1.58]	.016
** Potential moderator**	0.01	1.03	[1.00-1.06]	.093	0.00	1.00	[0.99-1.01]	.824
** Interaction term**	0.02	1.04	[1.00-1.07]	.048	0.01	1.01	[1.00-1.03]	.054
**Likelihood ratio test**				.050				.052
**App design**								
**Step 1**								
** Unstructured vs structured**	0.20	1.48	[1.27-1.74]	<.001	0.20	1.49	[1.26-1.75]	<.001
** Potential moderator**	0.02	1.03	[1.00-1.07]	.061	0.00	1.00	[0.99-1.02]	.715
**Step 2**								
** Unstructured vs structured**	0.20	1.48	[1.26-1.74]	<.001	0.20	1.48	[1.26-1.75]	<.001
** Potential moderator**	0.01	1.03	[1.00-1.07]	.080	0.00	1.00	[0.99-1.02]	.731
** Interaction term**	0.01	1.02	[0.99-1.06]	.252	0.01	1.01	[1.00-1.02]	.088
**Likelihood ratio test**				.254				.085
**Digital support**								
**Step 1**								
** Enhanced vs basic**	0.05	1.10	[0.88-1.37]	.417	0.04	1.09	[0.87-1.36]	.456
** Potential moderator**	0.02	1.03	[1.00-1.07]	.061	0.00	1.00	[0.99-1.02]	.717
**Step 2**								
** Enhanced vs basic**	0.05	1.10	[0.88-1.37]	.414	0.04	1.09	[0.87-1.37]	.445
** Potential moderator**	0.02	1.03	[1.00-1.07]	.058	0.00	1.00	[0.99-1.02]	.730
** Interaction term**	−0.00	1.00	[0.96-1.03]	.780	−0.00	1.00	[0.98-1.01]	.625
**Likelihood ratio test**				.780				.287

Dependent variable = number of modules completed. All models adjusted for age and gender. IRR = exp(2 * β), where β represents the unstandardized regression coefficient from effect-coded experimental factors. No interaction effects were statistically significant before adjustment for multiple comparisons.

Gender showed a significant main effect on engagement, with women completing more modules than men overall. Gender also significantly moderated the effect of app design on engagement. Among women, those assigned to the unstructured design completed 48% more modules than those assigned to the structured design (IRR = 1.48, 95% CI, 1.23-1.80, *P* < .001; Figure 2A). Engagement did not differ significantly by app design among men.

Caregiver age showed no significant main effect on engagement but significantly moderated the effects of both guidance and digital support. For every 10-year increase in age, WhatsApp-guided delivery (compared to self-guided use) was associated with a 15% increase in module completion (IRR = 1.15, 95% CI, 1.06-1.25, *P* = .001; Figure 2B). Similarly, enhanced digital support (compared to basic support) corresponded to a 15% increase in module completion for every 10-year increase in age (IRR = 1.15, 95% CI, 1.06-1.26, *P* = .002; Figure 2C). These findings suggest that although older caregivers were not more engaged overall, they benefited more from components involving additional support.

Two psychosocial factors significantly moderated the effects of design and delivery components on engagement. Although positive parenting was not associated with engagement overall, it significantly moderated the effects of both guidance and digital support. For guidance, each unit increase in positive parenting corresponded to a 3% increase in module completion in the WhatsApp-guided condition compared to self-guided use (IRR = 1.03, 95% CI, 1.02-1.04, *P* < .001; Figure 3A). In contrast, for digital support, each unit increase in positive parenting corresponded to a 4% decrease in module completion in the enhanced support condition compared to basic support (IRR = 0.96, 95% CI, 0.95-0.98, *P* < .001; Figure 3B).

Depressive symptoms similarly moderated the effect of guidance. Although depressive symptoms were not associated with engagement overall, each unit increase in symptoms corresponded to a 6% increase in module completion in the WhatsApp-guided condition compared to self-guided use (IRR = 1.06, 95% CI, 1.03-1.09, *P* < .001; Figure 3C). No significant moderation effects were observed for child maltreatment.

## Discussion

To our knowledge, this study is the first to examine how caregiver characteristics moderate the effects of discrete design and delivery components on engagement with a digital parenting intervention in an LMIC. It reports moderation analyses from a factorial experiment conducted in Tanzania as part of a MOST optimization trial, with main component effects reported elsewhere.^19^ Building on findings that WhatsApp guidance and an unstructured app design significantly improved engagement, this study identifies for whom specific components were most effective.

### Overall findings

Gender, age, positive parenting, and depressive symptoms moderated the effects of specific design and delivery components on engagement with ParentApp. These findings suggest that tailoring app design and delivery to specific caregiver characteristics may further enhance engagement in digital parenting interventions. In contrast, financial stress, food insecurity, and child maltreatment did not significantly moderate the effects of any tested component, suggesting that some implementation strategies may operate similarly across families experiencing socioeconomic and parenting-related adversity. The following sections discuss each significant moderator in turn.

Gender moderated the effect of app design, with women showing significantly higher engagement when assigned to the unstructured version, whereas no significant differences in engagement by app design were observed among men. This version differed not only in navigation style but also in its visual design, featuring culturally adapted illustrations that predominantly depicted women. These features may have increased the perceived relevance or acceptability of the intervention for women. However, because visual content and navigation structure were bundled within the same component, the specific mechanisms underlying this effect cannot be isolated. Nevertheless, the findings highlight the importance of gender-transformative design.

Prior research, including a systematic review of father-inclusive interventions in LMICs, identified multiple barriers to male caregiver engagement, including time constraints, restrictive norms around caregiving, and lack of targeted design features.^45^ Programs such as Parenting for Respectability, an in-person intervention in Uganda, have demonstrated the feasibility of gender-transformative approaches such as men-only introductory sessions and content affirming fatherhood to engage male caregivers while challenging restrictive gender norms.^46^ In digital contexts, qualitative research on the chatbot-based ParentText program in South Africa and Jamaica found that anonymity, mobile delivery, and equitable messaging helped fathers feel more included and supported.^47^ Together, these findings suggest that both content and delivery format play important roles in shaping the accessibility and relevance of parenting interventions for men.

Caregiver age moderated the effects of both guidance and digital support, with older caregivers showing higher engagement when assigned to either WhatsApp guidance or enhanced digital support. Although age was not associated with engagement overall, these findings suggest that older caregivers may benefit more from components that incorporate additional support. Field reports indicated that many older participants relied on borrowed smartphones and had limited digital experience. In such contexts, opportunities to ask questions and receive support may have improved engagement by addressing barriers related to digital competence and confidence. This interpretation aligns with a systematic review of attrition in digital health apps, which identified limited technical skills and lack of prior digital experience as key contributors to disengagement.^24^ A further systematic review found that tailoring digital interventions to users’ digital literacy needs can enhance engagement, particularly among older adults.^25^ Together, these findings highlight the value of integrating support strategies that address digital literacy, particularly for older caregivers. Programs may benefit from offering optional, low-intensity supports such as onboarding assistance or peer and facilitator support as part of standard delivery. As digital parenting interventions scale, automated approaches such as interactive onboarding tools may offer a more sustainable alternative. Research with older adults suggests that automated real-time, trial-and-error onboarding support can improve smartphone use, confidence, and motivation more effectively than static instructions,^48^ although the feasibility of such approaches within parenting interventions remains to be tested.

Positive parenting practices moderated engagement in opposite directions across 2 components. For the guidance component, higher baseline positive parenting was associated with greater module completion in the WhatsApp-guided condition. One possible explanation is that WhatsApp groups reinforced existing parenting strengths through structured reflection and peer exchange, thereby sustaining motivation to practice and refine skills over time. This interpretation aligns with evidence that guided digital delivery can enhance parenting-related self-efficacy.^22^^,^^23^

In contrast, caregivers with higher baseline positive parenting showed lower engagement when assigned to enhanced digital support. These caregivers may have perceived less need for additional onboarding support, potentially contributing to lower engagement with this component.^32^ From a self-determination theory (SDT) perspective, individuals with stronger perceived competence may disengage when support feels overly directive or undermines autonomy.^49^ Allowing caregivers to select their preferred level of digital support may therefore better align with individual motivation and perceived capacity. However, preference alone may not fully reflect actual digital literacy needs. Future research is needed to determine whether preference-based, assessment-based, or hybrid approaches most effectively balance autonomy, need, and engagement.

Caregiver depressive symptoms also moderated engagement, with caregivers reporting higher symptom levels completing more modules when assigned to the WhatsApp-guided condition. Prior research has linked parental psychological distress to lower engagement in self-directed digital parenting programs.^33^ More broadly, depressive symptoms have been identified as barriers to participation in digital mental health interventions,^25^ potentially due to low mood, fatigue, or reduced motivation. In the context of this study, facilitator-moderated WhatsApp groups may have mitigated these challenges by fostering social support and peer connection, both of which have been identified as facilitators of engagement in digital interventions.^25^ These findings suggest that delivery approaches incorporating social connection may be particularly beneficial for caregivers experiencing elevated depressive symptoms.

### Limitations

Several limitations should be acknowledged when interpreting these findings. First, although the trial was adequately powered to detect main effects, moderation analyses typically require substantially larger sample sizes, particularly when moderators are unevenly distributed. As is common in moderation research, these findings should therefore be interpreted as exploratory.^31^ Future optimization trials should conduct power calculations specifically for interaction effects.

Second, all moderation analyses were exploratory and included a broad set of candidate moderators, reflecting the limited prior evidence base in digital parenting research, particularly in LMIC contexts. Although corrections for multiple comparisons were applied to reduce the risk of false positives, future work should refine theoretical models of engagement to guide more targeted moderator selection. In addition, child characteristics such as age, gender, and behavior were not examined but may play an important role in shaping caregiver engagement.

Third, although measures were drawn from widely used open-access instruments and underwent cultural adaptation and pilot testing, several moderators were assessed using brief or single-item measures. Internal consistency was modest for some multi-item scales, introducing some degree of measurement error. More comprehensive assessments would strengthen confidence in these findings.

Fourth, engagement was operationalized solely as module completion. Although module completion is one of the most widely used and comparable indicators of engagement in digital parenting research,^36^ it does not capture dimensions of engagement quality such as depth of participation and skill enactment.^12^ Future studies should incorporate multidimensional engagement metrics to provide a more comprehensive understanding of engagement quality.

Fifth, the sample was drawn exclusively from low-income communities, which may have limited variability in socioeconomic indicators and reduced sensitivity to detect moderation by factors such as financial stress or food insecurity. Replication in more socioeconomically diverse samples would strengthen confidence in these findings. Finally, the study was conducted under pragmatic implementation conditions, including community-led recruitment, variable smartphone access, and variation in facilitation practices across clusters. While this enhances ecological validity and real-world relevance, it may also have introduced additional variability. Future implementation research should examine how differences in delivery context and fidelity influence both engagement and intervention outcomes.

### Implications

This study offers several implications for the adaptation and implementation of digital parenting interventions. First, the absence of moderation effects for financial stress, food insecurity, and child maltreatment suggests that the tested components functioned similarly across families experiencing socioeconomic and parenting-related adversity. Although null findings are often underreported, their consistency here is notable. In particular, the findings suggest that the design and delivery components examined here did not appear to disadvantage caregivers experiencing higher levels of adversity. This interpretation aligns with findings from the in-person PLH Teens RCT in South Africa, which similarly reported no significant moderation of outcomes by caregiver or household risk.^31^

Second, the identification of moderation by gender, age, positive parenting practices, and depressive symptoms suggests potential value in tailoring design and delivery features to enhance engagement. These findings align with precision health approaches, which emphasize adapting interventions to individual characteristics.^50^ Although this study relied on static baseline characteristics to inform tailoring, future efforts may benefit from incorporating more dynamic data sources. Advances in smartphone-based monitoring, wearable sensors, and AI-enabled platforms offer opportunities to integrate real-time behavioral, contextual, and physiological data into adaptive delivery systems.^51^^,^^52^ Although such technologies are not yet widely implemented in LMIC settings, they represent a promising direction for developing more responsive and scalable parenting support.

Third, this study illustrates how the MOST framework, and specifically factorial experiments, can be used to examine both overall component effectiveness and subgroup differences within a pragmatic LMIC context. These findings contribute to growing evidence that optimization approaches can support more contextually responsive and scalable intervention delivery.^18^^,^^53^ For intervention developers and implementers, the trial provides an applied example of how such methods can inform delivery refinement. More broadly, incorporating optimization approaches into intervention development may support more inclusive, targeted, and sustainable digital health delivery systems.

## Conclusion

This study provides novel evidence from an LMIC context that caregiver characteristics may moderate the effects of specific design and delivery components on engagement with a digital parenting intervention. Guided delivery and enhanced digital support appeared particularly beneficial for older caregivers, while guided delivery was also associated with higher engagement among caregivers with more positive parenting practices at baseline and those reporting higher depressive symptoms. The unstructured app design was more effective for women. At the same time, the absence of moderation by financial stress, food insecurity, and child maltreatment suggests that the tested components operated similarly across families experiencing socioeconomic and parenting-related adversity. By identifying for whom specific design and delivery components are most beneficial, this study contributes to emerging efforts to develop more person-centered and contextually responsive digital parenting interventions. As such programs continue to scale in LMIC settings, integrating optimization methods into intervention development may help ensure that implementation strategies are not only effective on average, but also responsive to the needs of diverse caregiver groups.

## Supplementary Material

kaag033_Supplementary_Data

## Data Availability

De-identified data from this study are not available in a public archive. De-identified data from this study will be made available by emailing the corresponding author.

## Analytic plan pre-registration

The analysis plan was not formally pre-registered.

## Analytic code availability

Analytic code used to conduct the analyses presented in this study are not available in a public archive. They may be available by emailing the corresponding author.

## Materials availability

Materials used to conduct the study are available from the corresponding. ParentApp is freely available and open source online.
