# Supplementary material for: Moderators of design and delivery component effects on engagement with a digital parenting intervention: evidence from a factorial randomized trial in Tanzania
Source: Ann Behav Med. 2026 Jun 21;60(1):kaag033. doi: 10.1093/abm/kaag033 (PMC13283430; doi:10.1093/abm/kaag033)
Supplement: kaag033_Supplementary_Data [file kaag033_supplementary_data.docx]

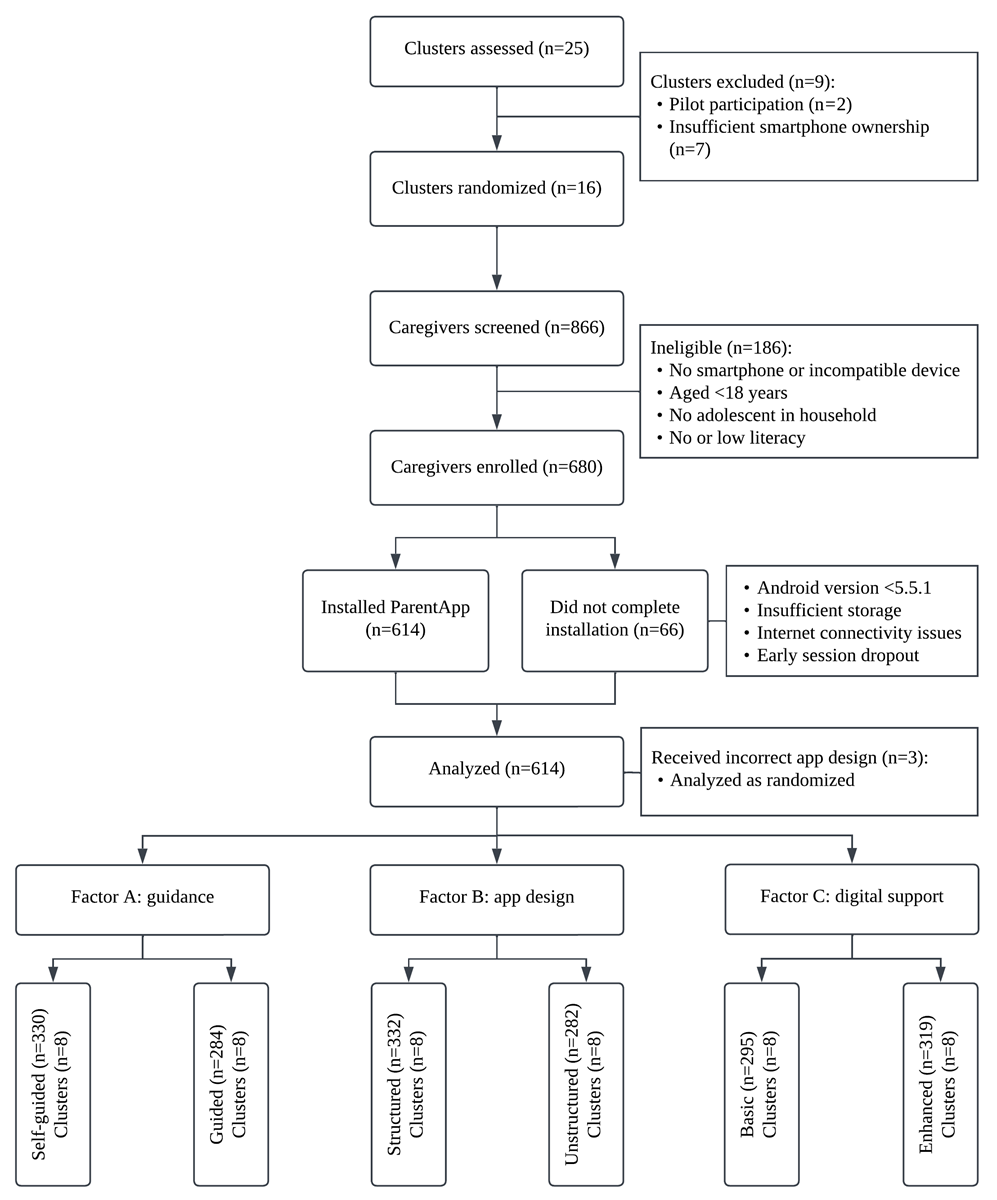


**Supplementary Figure S1.** CONSORT (Consolidated Standards of Reporting Trials) diagram of participant flow through the 2×2×2 factorial trial.
